# Borderline Personality Disorder, Childhood Trauma, and Suicide Attempts: A Retrospective Study at Ar-razi University Hospital, Morocco

**DOI:** 10.1192/j.eurpsy.2026.11905

**Published:** 2026-07-31

**Authors:** O. Seyar, L. Azizi, A. Zaki, S. Belbachir, A. Ouanass

**Affiliations:** psychiatry, arrazi hospital, salé, Morocco

## Abstract

**Introduction:**

Borderline Personality Disorder (BPD) is strongly associated with emotional instability, impulsivity, and a high risk of suicide. Childhood trauma has been identified as a major factor contributing to suicidal behaviors in these patients. This study aimed to describe the sociodemographic profile, trauma history, and characteristics of suicide attempts among Moroccan patients with BPD, and to explore the relationship between childhood trauma and suicidal behavior.

**Objectives:**

To analyze the association between childhood traumatic experiences and suicide attempts in patients with BPD hospitalized at Ar-razi University Psychiatric Hospital, Salé.

**Methods:**

We conducted a retrospective, descriptive, and analytical study from September 2022 to September 2024, including 66 medical records of patients diagnosed with BPD according to DSM-5 criteria. Sociodemographic data, personal and family history, and childhood trauma were assessed. Suicide attempts were evaluated using the Beck and Pierce Suicide Severity Scales. Multiple regression analysis was applied to identify predictors of suicide attempts. Statistical analysis was performed using SPSS, with a significance threshold of 0.05.

**Results:**

Among BPD patients, a high prevalence of childhood trauma was identified. Most suicide attempts (72%) occurred in a depressive context. The severity of suicide attempts, as measured by Beck and Pierce scales, was significantly associated with childhood trauma. Regression analysis revealed that early traumatic experiences were significant predictors of the number and severity of suicide attempts. Our findings confirm the strong association between childhood trauma and suicidal behaviors in BPD, consistent with international literature. Despite limitations related to retrospective design and sample size, the results underline the importance of screening for trauma in clinical practice.

**Conclusions:**

Childhood trauma is a major risk factor for suicide attempts in BPD. Early identification and preventive strategies are essential to reduce suicidal risk in this vulnerable population.

**Disclosure of Interest:**

None Declared

